# Epigenetic checkpoint blockade: new booster for immunotherapy

**DOI:** 10.1038/s41392-021-00707-z

**Published:** 2021-07-22

**Authors:** Ziqiao Wang, Bingjing Wang, Xuetao Cao

**Affiliations:** 1 Department of Immunology, Institute of Basic Medical Sciences, Peking Union Medical College, Chinese Academy of Medical Sciences, Beijing, China; 2 Laboratory of Immunity and Inflammation, College of Life Sciences, Nankai University, Tianjin, China

**Keywords:** Immunotherapy, Molecular medicine

In a recent study published in Nature, Griffin et al. reported that blockade of SETDB1 enhances anti-tumor immune response, shedding new light on the potential epigenetic therapy to specifically improve immune checkpoint blockade (ICB) effectiveness.^1^

ICB therapy based on the monoclonal antibodies targeting PD-1/PD-L1 and CTLA-4 has achieved the remarkable clinical benefits in patients with different malignancies. Although ICB has been considered as one of the most pioneering treatments in last decade, the challenges of ICB still persist due to the low response and recurring resistance observed in cancer patients. Therefore, the molecular mechanisms of resistance to checkpoint inhibitors need to be further investigated to design the potential combination strategies of immunotherapy. Epigenetic regulation, including DNA methylation, histone modification, and chromatin remodeling, plays important roles in cancer progression.^2^ Methylation profile has been recognized as a vital index in diagnosis and prognosis of hematological malignancies.^3^ In addition, epigenetic remodeling is essential for the regulation of immunity and inflammation.^4^ Increasing reports have shown that epigenetic modulators can reprogram tumor microenvironment (TME) to increase the susceptibility of tumor cells to cytotoxic T-cell killing, leading to the enhanced anti-tumor immune response.

To identify epigenetic regulators that control anti-tumor responses to ICB, Griffin et al. performed a curated in vivo CRISPR screening by transplanting B16 or LLC cells transduced with sgRNA library targeting 936 genes associated with chromatin regulation into immunodeficient mice and C57BL/6J mice with or without ICB treatment, followed by direct readout of the depleted sgRNA ranking. The authors discovered that *Setdb1* was the top-ranked sensitizer in both models and *Setdb1* deficiency robustly inhibited tumor growth in ICB treated rather than untreated mice. Consistent with mice models, *SETDB1* amplification or overexpression in human tumors inversely correlated with immune signatures and overall survival according to TCGA database. Notably, upregulation of *SETDB1* indicated the worse therapeutic effect of PD-1 blockade but not mTOR inhibitor in renal cancer patients.

**Fig. 1:**
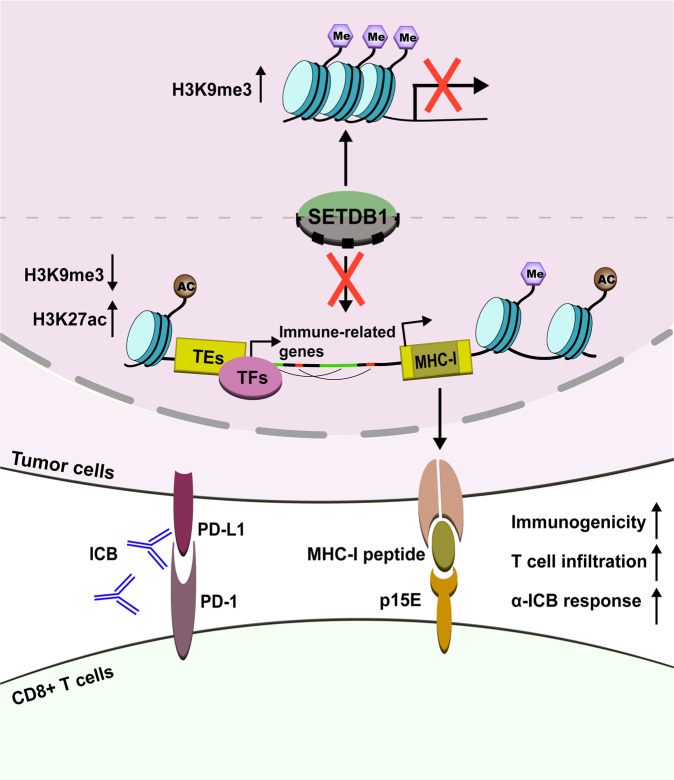
The schematic of SETDB1 repression in promoting the cytotoxic T-cell responses against tumor. Inhibition of SETDB1 induces extensive TEs expression and segmental duplications that not only activate immune-related genes but also encode MHC-I peptides as neoantigens, leading to the enhanced immunogenicity and killing susceptibility of tumor cells. Me methylation, Ac acetylation, TEs transposable elements, TFs transcriptional factors, ORF open reading frame, ICB immune checkpoint blockade

SETDB1, a H3K9 lysine methyltransferase, involves in transcriptional silencing of multiple genes at specific loci and therefore plays essential roles in early development and carcinogenesis of various tissues. By deciphering the synergetic results of various sequencing analysis including ChIP-seq, RNA-seq, and ATAC-seq, Griffin et al. found that SETDB1-dependent H3K9me3 peaks were significantly amassed in the open genome and large domains, and SETDB1 domains were enriched for transposable elements (TEs) of the long terminal repeat (LTR) family and segmental duplications which are related with immune processes, especially defense response to virus, antigen presentation, complement activation, and T-cell cytotoxicity. While in *Setdb1*-deficient cells, chromatin accessibility, and specific enhancer marker H3K27 acetylation harmoniously augmented at distal TEs within SETDB1 domains which could further activate nearby immune-related genes regulated by cell-specific transcription factors, for instance, CTCF and JUN-AP-1. Such segmental duplications often emphasized a dominant role for genetic evolving and structure variation, prompting that segmental duplications and TEs regulated by SETDB1 were latent regulator of immune gene expression within evolving loci (Fig. 1).

Notably, the authors discovered bi-directional transcriptions of induced TEs were too rare to generate viral mimicry or activate antiviral pathways. Instead, numerous TEs especially LTR family encoding intact viral open reading frames such as murine leukemia virus (MuLV) envelope protein were detected in *Setdb1* knockout cells. Further proteomics results revealed that TE-encoded MHC-I peptides are predicted targets of adaptive immune response under *Setdb1* suppression. Similarly, they found that *SETDB1* knockout in human cancer cells induced TE-encoded MHC-I peptide but not viral mimicry. The authors next performed T-cell receptor (TCR) repertoire and scRNA-seq combined transcriptional profiling to depict immune infiltration in TME. *Setdb1* deficiency resulted in the expansion of tumor-infiltrating cytotoxic CD8^+^ T cells and p15E-tetramer^+^ T cells as along with the upregulated genes related with cytotoxicity among p15E-specific TCR, which is known to initiate MuLV-derived MHC-I peptide for stimulating antineoplastic T-cell responses. MHC-I ablation by knockout *B2m* or CD8^+^ T cells exhaustion by specific antibody both impaired ICB sensitivity in *Setdb1* knockout cells. Together, MHC-I-associated antigen presentation and CD8^+^ T-dependent antigen recognition are the key factors to enhance anti-tumor immune response by SETDB1 suppression.

Yet, several questions remain to be solved. Although SETDB1 has similar function in mice and human, its targeting genomic loci may be context different therefore needs specific definition. It is necessary to testify the potential efficacy of SETDB1 suppression in more tumor models. Also, further investigation is needed to clarify whether the enhancing effect of SETDB1 modulator is universal or ICB specific. In addition, it is unclear whether SETDB1 affects TEs, segmental duplications, and immune genes expression in stromal cells. Besides cytotoxic T cells, SETDB1 has been reported to play vital roles in other immune cells as well as tumor cells. For example, SETDB1 stabilizes Th2 cells by blocking ERVs that shape the Th1 gene network while promotes cancer metastasis by multiple mechanisms. It is interesting to demonstrate the effect on the panorama of TME by blocking SETDB1.

In conclusion, blockade of SETDB1 could enhance specific cytotoxic T-cell responses against tumor by not only activating immunostimulatory genes but also encoding retroviral antigens and generating MHC-I peptides as neoantigens, proposing SETDB1 as a highly potential target to synergize with ICB therapy. Recent studies have reported multiple ongoing clinical trials combining epigenetic therapies with immunotherapies, such as HDAC inhibitors, DNMT inhibitors, LSD1 inhibitors and EZH2 inhibitors which could lead to more potent CD8^+^ T cell anti-tumor immune responses. The specific targeting of SETDB1 has not been described. However, mithramycin, a clinically anti-tumor antibiotic, has been reported to nonspecifically suppress SETDB1 expression in a dose-dependent manner, which binds to GC-rich regions of genome thus interferes with the association of transcriptional activators at *Setdb1* promoters.^5^

Despite the combination of epigenetic modulator with immunotherapy is promising to achieve more effective responses, it is crucial to identify the appropriate patient population. Therefore, extensive analyses of clinical samples by high-throughput sequencing, single-cell technology along with whole-genome scanning are indispensable to provide more accurate information for designing the personalized treatment strategy. Besides, considering the pleiotropic and contextual immune phenotypes associated with SETDB1, the possible adverse effects of SETDB1 inhibitor still worth of attention.
